# iTRAQ-Based Quantitative Proteomics Indicated Nrf2/OPTN-Mediated Mitophagy Inhibits NLRP3 Inflammasome Activation after Intracerebral Hemorrhage

**DOI:** 10.1155/2021/6630281

**Published:** 2021-02-09

**Authors:** Yijun Cheng, Mingjian Liu, Hao Tang, Bin Chen, Guoyuan Yang, Weiguo Zhao, Yu Cai, Hanbing Shang

**Affiliations:** ^1^Department of Neurosurgery, Rui Jin Hospital, Shanghai Jiao Tong University School of Medicine, Shanghai 200025, China; ^2^Neuroscience and Neuroengineering Research Center, Med-X Research Institute, Shanghai Jiao Tong University, Shanghai 200030, China; ^3^Department of Neurology, Rui Jin Hospital, Shanghai Jiao Tong University School of Medicine, Shanghai 200025, China; ^4^Department of Neurosurgery, North Rui Jin Hospital, Shanghai Jiao Tong University School of Medicine, Shanghai, China

## Abstract

Intracerebral hemorrhage- (ICH-) induced secondary brain injury (SBI) is a very complex pathophysiological process. However, the molecular mechanisms and drug targets of SBI are highly intricate and still elusive, yet a clear understanding is crucial for the treatment of SBI. In the current study, we aimed to confirm that nuclear factor-E2-related factor 2 (Nrf2)/Optineurin- (OPTN-) mediated mitophagy alleviated SBI by inhibiting nucleotide-binding oligomerization domain-like receptor pyrin domain-containing 3 (NLRP3) inflammasome activation based on the isobaric tag for relative and absolute quantization (iTRAQ) quantification proteomics. Human ICH brain specimens were collected for iTRAQ-based proteomics analysis. Male Nrf2 wild-type (WT) and knockout (KO) mice were employed to establish ICH murine models. The survival rate, hematoma volume, neurofunctional outcomes, blood-brain barrier (BBB) permeability, brain edema, spatial neuronal death, NLRP3 inflammasome, inflammatory response, mitochondrial function, and mitophagy level were evaluated after ICH. The iTRAQ quantification analysis showed that the differentially expressed proteins (DEPs), Nrf2 and NLRP3, were closely associated with the initiation and development of SBI after ICH. The Nrf2 KO mice had a significantly lower survival rate, bigger hematoma volume, worse neurological deficits, and increased BBB disruption, brain edema, and neuronal death when compared with the Nrf2 WT mice after ICH. Furthermore, Nrf2 KO enhanced NLRP3 inflammasome activation and neuroinflammation as evidenced by the NF-*κ*B activation and various proinflammatory cytokine releases following ICH. Moreover, Nrf2 could interact with and modulate the mitophagy receptor OPTN, further mediating mitophagy to remove dysfunctional mitochondria after ICH. Furthermore, OPTN small interfering RNA (siRNA) increased the NLRP3 inflammasome activation by downregulating mitophagy level and enhancing mitochondrial damage in the Nrf2 WT mice after ICH. Together, our data indicated that Nrf2/OPTN inhibited NLRP3 inflammasome activation, possibly via modulating mitophagy, therefore alleviating SBI after ICH.

## 1. Introduction

Intracerebral hemorrhage (ICH) is a potentially life-threatening stroke subtype with the highest morbidity and mortality rate. This critical disorder results in a monthly mortality rate of 33.33% and often causes residual disability, high risk of recurrence, and different kinds of neurofunctional complications such as dementia and epilepsy [1]. It is reported that ICH affects about 2 million people each year in all the world [2]. Although operation is widely used in various forms as potentially life-saving in clinic, its therapeutic effect remains controversial [3, 4]. Still, no clearly proven medical treatment for ICH exists. In addition to the physical hematoma compression causing primary brain injury after ICH onset immediately, the pathophysiological responses caused by the cytotoxic extravasated blood components contribute to secondary brain injury (SBI) [5]. Considering that SBI after ICH is a very complex pathophysiological process, using a comprehensively effective approach to investigate the intricate pathophysiological molecular mechanisms is especially important. Nowadays, advances in proteomics technologies have enabled researchers to identify differentially expressed protein (DEP) profiles and to further explore their potential mechanisms. Isobaric tag for relative and absolute quantization (iTRAQ) is a mature technique widely used in proteomics which can quantify proteins based on peptide labeling and allows the identification and quantification of peptides and proteins from various samples within broad dynamic ranges of protein abundance [6]. Thus, it is highly feasible to find the target DEPs of SBI after ICH and reveal the underlying molecular mechanisms via the iTRAQ method.

As a member of the cap‘n'collar (CNC) family of the basic region leucine zipper transcription factor, nuclear factor-E2-related factor 2 (Nrf2) can induce a group of cytoprotective genes containing “antioxidant response element (ARE)” at their promotors, which function to scavenge reactive oxygen species (ROS), suppress inflammation and oxidative stress, preserve xenobiotic metabolism, detoxify, maintain autophagy level, etc., to restore cellular homeostasis [7]. Indeed, our prior data have proven that Nrf2 was upregulated in kinds of ICH animal models and could counteract oxidative insults as an adaptive response [8–10]. When the Nrf2 gene was knocked out, the animals had elevated hemorrhage volume, leukocyte infiltration, and oxidative insults after ICH surgery [11]. Given that inflammation can be triggered by oxidative stress, Nrf2 has been well reported that it controls inflammatory response effectively in different kinds of inflammatory and neurodegenerative diseases.

Inflammasomes trigger and provide a platform for the downstream inflammatory cascade reaction. Inflammasomes were defined by their sensor proteins, namely, pattern-recognition receptors (PRRs), which can oligomerize to form an activating pro-caspase-1 platform in response to various harmful stimuli, including oxidative stress. So far, several members of PRPs have been verified, such as leucine-rich repeat- (LRR-) containing protein (NLR) family members NLRP1, NLRP2, NLRP3, NLRC4, NLRP6, absent-in-melanoma 2 (AIM2), etc. Among these PRPs, NLRP3 is the best and most extensively characterized. NLRP3 can connect to caspase-1 via apoptosis-associated speck-like protein containing a caspase recruitment domain (ASC) to constitute a large molecular complex called the NLRP3 inflammasome, which further mediates the maturation and release of several proinflammatory cytokines, including interleukin-1beta (IL-1*β*) and interleukin-18 (IL-18) [12]. Until now, the NLRP3 inflammasome has been documented to participate in kinds of murine brain injury models, including ischemic stroke [13], traumatic brain injury [14], and subarachnoid hemorrhage [15]. Notably, our prior studies have also proven that the NLRP3 inflammasome was time-dependently activated and amplified neuroinflammation and SBI after ICH [16]. Later, our study also found that interference with the NLRP3 inflammasome by exogenous drugs such as cordycepin [16] and ghrelin [10] effectively inhibited neuroinflammation and SBI following ICH, indicating that NLRP3 inflammasome is a promising therapeutic target for SBI post-ICH. Given the sufficient evidence of the role of oxidative stress and neuroinflammation in the brain injury after ICH, Nrf2 and NLRP3 inflammasome may be the prospective breakthrough in the treatment of SBI.

Mitochondrial dysfunction has been shown to play a crucial role in the activation of NLRP3 inflammasome [17]. Mitochondrial dysfunction includes three major aspects: (i) decreased mitochondrial respiratory chain oxidative phosphorylation (OXPHOS) activity, (ii) increased mitochondrial ROS (mtROS) production, and (iii) elevated mitochondrial DNA (mtDNA) damage. When the OXPHOS activity decreases, the direction of electron transfer changes, resulting in excessive ROS generation. Increased mtROS can not only lead to the opening of membrane permeability transition hole which further allows mtDNA translocation into the cytoplasm but also cause the mtDNA copy number reduction and mutation. In turn, mtDNA damage further increases OXPHOS activity abnormality and mtROS generation. All the ROS, mtROS, and abnormal mtDNA can activate the NLRP3 inflammasome. Thus, finding potential strategies for removing the damaged mitochondria can effectively halt the NLRP3 inflammasome activation after ICH.

Selective elimination of dysfunctional mitochondria by autophagy, namely, mitophagy, is the main mechanism of cellular mitochondrial quality control. One of the central steps of mitophagy is the formation of mitophagosome. Optineurin (OPTN) is a multifunctional ubiquitin-binding autophagy receptor which can bind to ubiquitinated cargo through ubiquitin-binding domains (UBD) and then link cargo to mitophagosomal membranes mainly via binding to LC3 through a short peptide sequence well known as the LC3-interacting region (LIR). The LIR motif within OPTN is a central feature of the canonical mitophagy model [18]. In addition, OPTN has also been documented to promote the recruitment of the unc51-like activating kinase 1/2 complex (ULK1/2-Atg13-FIP200-Atg101) during the PINK1/Parkin-mediated mitophagy [19]. Therefore, OPTN-mediated mitophagy may effectively clear the damaged mitochondria and inhibit subsequent NLRP3 inflammasome activation after ICH.

Based on the above-mentioned premises, the current study was designed to reveal the total DEP profiles and aimed at exploring the potentially underlying mechanisms of ICH via the iTRAQ method. According to the proteomics results, we further revealed that the target DEPs, Nrf2/OPTN-mediated mitophagy suppressed the neuroinflammation via inhibiting NLRP3 inflammasome activation, thereby alleviating SBI following ICH.

## 2. Materials and Methods

### 2.1. iTRAQ Quantification Analysis

Human brain samples were collected from ICH patients who were transferred to our hospital for surgical treatment. During the operation, the broken perihematomal brain tissues were collected as the ICH group, while the corresponding normal brain tissues of the control group were collected from patients with epilepsy as their first symptom underwent surgery for non-ICH vascular diseases, including cavernous hemangioma and arteriovenous malformation. Proteins were extracted from the above-mentioned samples with protein lysis buffer. The supernatants were centrifuged and digested with alkylate and trypsin at 37°C overnight. Afterward, tags were employed to label the peptides according to the technical manuals of the iTRAQ Reagents 8-plex kits (AB Sciex, Foster City, CA, USA). The HPLC system (Shimadzu, Kyoto, Japan) was employed to conduct the iTRAQ-labeled peptides for strong cation exchange (SCX) chromatography. These peptides were reconstituted with buffer A (20 mM HCOONH_4_, pH 10) and then placed on a Gemini-NX C18 SCX column (3 mm, 2∗150 mm, Phenomenex) with buffer B (20 mM HCOONH_4_ 80% acetone pH 10). The elution absorbance was monitored at 214 nm/280 nm. The flow rate was set at 200 ml/min. Eluted fractions were collected as 24 fractions and vacuum-concentrated. For subsequent LCMS/MS, final fractions were reconstituted in 50% trifluoroacetic acid.

The peptides were analyzed by a Q Exactive Mass Spectrometer (Thermo Fisher, Waltham, USA) and identified using a Thermo Dionex Ultimate 3000 RSLC nanosystem (Thermo Fisher, Waltham, USA). The peptide mixture was then isolated from the PepMap C18 RP column (2 mm, 75 mm∗150 mm, 100 A) in the binary buffer system which consisted of 0.1% formic acid (buffer A) and 84% CAN in 0.1% formic acid (buffer B) under a total of 65 min linear gradient of 4-90% buffer B at 300 nl/min flow rate. Then, the target peptides were measured by using Q Exactive online and MS data were acquired through a top 20 mode and high-energy collisional dissociation (HCD) method. According to the most chosen abundant precursor ions, the scan range was set at 350-1800 m/z. The dynamic exclusion was set as 40 s. The automatic gain control (AGC) target value was 3E6, and the underfill ratio was defined as 0.1%.

Afterward, the above acquired raw data were searched via ProteinPilot Software (AB Sciex, Foster City, CA, USA; version 5.0.1) based on the UniProt rat database. The search parameters were set as the following: iTRAQ 8 plex peptide for sample type and trypsin for digestion enzyme. Biological modifications were chosen as ID focus. To minimize the false-positive identification, only proteins with a confidence threshold cutoff more than 1.3 and protein confidence = 95% were chosen as the differentially expressed proteins (DEPs). The data were further based upon a false discovery rate (FDR) ≤ 1% confidence for protein identification. For the next proteomics bioinformatics analysis, functional classification and Gene Ontology (GO) enrichment analysis were constituted by DAVID Bioinformatics Resources v6.8 (https://david.ncifcrf.gov/). DEPs were further classified using GO category (http://www.geneontology.org), including the biological process (BP), cellular compartments (CC), and molecular function (MF). Target pathway analyses were identified with Kyoto Encyclopedia of Genes and Genomes (KEGG) (https://www.genome.jp/kegg/). The heat map of DEPs was generated by using MeV 4.9 software. The functional protein-protein interaction (PPI) network was explored in STRING (http://string-db.org/).

### 2.2. Animals and Experimental Groups

Nrf2 knockout (KO) ICR mice were kindly originally provided by Dr. Thomas W. Kensler (Johns Hopkins University, Baltimore, MD, USA). Nrf2 wild-type (WT) male ICR mice, 6- to 8-week-old, were purchased from the Experimental Animal Center of the Chinese Academy of Sciences (Shanghai, China). The current study was divided into two parts of the experimental design. Part 1: to observe the effect of Nrf2 on the SBI and NLRP3 inflammasome inhibition, animals were randomly divided into 4 groups: (i) Nrf2 WT sham group (*n* = 36), (ii) Nrf2 KO sham group (*n* = 36), (iii) Nrf2 WT ICH group (*n* = 48), and (iv) Nrf2 KO ICH group (*n* = 48). Part 2: to investigate whether Nrf2 inhibited NLRP3 inflammasome activation via OPTN-mediated mitophagy, a total of 36 Nrf2 WT mice were equally divided into 3 groups: (i) vehicle group, (ii) si-NC group, and (iii) si-RNA-OPTN group. A total of 5 *μ*l volume of siRNA OPTN (500 pmol) and si-NC (GenePharma, Shanghai, China) were injected intracerebroventricularly 48 h preoperation. All animal procedures were approved by the Institutional Animal Care and Use Committee of Shanghai Jiao Tong University, Shanghai, China.

### 2.3. ICH Model Induction

As we previously described [10], the autologous blood infusion was injected into the striatum stereotactically to mimic clinical ICH onset. Briefly, animals were deeply anesthetized with ketamine/xylazine (100/10 mg/kg, Sigma-Aldrich, St. Louis, MO, USA) intraperitoneally. Then, mice were fixed on a stereotactic frame (RWD Life Science Co., Shenzhen, China). A dental drill engine was employed to drill a 1 mm diameter bone hole which was 2.3 mm lateral to the midline and 0.2 mm anterior to the bregma. A 25 *μ*l volume of nonanticoagulated autologous blood was collected from the mouse tail vein. The syringe needle was advanced exactly 3.0 mm into the right basal ganglia carefully. The whole blood was injected with a microinfusion pump (WPI, Sarasota, FL, USA) in two stages. Firstly, an initial 5 *μ*l volume of blood was injected at 2 *μ*l/min. With a 5 min interval, the remaining 20 *μ*l blood was injected at 2 *μ*l/min again. The needle was left in place for an additional 10 min postinjection to prevent the possible blood backflow. After removal of the needle, the burr hole was sealed with bone wax and the scalp was sutured.

### 2.4. Hematoma Volume

Mice were euthanized at 3 d after operation. The brain samples were rapidly harvested, frozen, and cut into 4 *μ*m pieces. Then, the sections were stained with crystal violet. The stained sections were photographed and quantified with ImageJ software. The results were presented as the amounts of the lesion area multiplied by the interslice distance.

### 2.5. Neurobehavioral Deficits

Neurological function was tested at 3 days after operation by investigators who were blinded to the divided groups. All procedures were performed as we previously reported [10]. Briefly, for the beam walking test, mice were placed midway on a 90 cm long, 1.5 cm wide, and 40 cm high rod with two platforms at both ends. To avoid falling, mice moved along the horizontal rod to reach anyone of the platforms. The scores were recorded by measuring the distance and time of mice to fall as we previously described [10]. For the corner turn test, mice were left in a 30° corner. To exit, mice will turn either left or right. The percentages of turning left were recorded. For the forelimb placing test, the mouse vibrissae were stimulated to observe the respective response of left paw placing. The percentages of left paw placing were recorded. For the Rotarod test, mice were trained three times a day for a total of 3 d presurgery. Each trial persisted for 5 min with a 2 h rest interval. According to the animals' habitation, the trail speed accelerated gradually from 4 to 40 rpm. After ICH induction, mice were still trained as before and the latency to fall was recorded on day 3.

### 2.6. Evans Blue Extravasation

Evans blue extravasation assay was performed to assess the permeability of the blood-brain barrier (BBB) after ICH. In brief, a 2% Evans blue (4 ml/kg, Sigma, St. Louis, MO, USA) solution in saline was administered into the mice tail vein 3 h before sacrifice on day 3 after operation. After 3 h circulation, animals were deeply anesthetized and perfused with cold saline through the left ventricle until the colorless fluid outflowed from the right atrium. Afterward, the ipsilateral and contralateral hemispheres were rapidly collected. Then, the hemispheres were weighed and homogenized in 1 ml of 50% ice-cold trichloroacetic acid (Sigma, St. Louis, MO, USA). After centrifugation, the supernatant was collected and mixed with ethanol (1 : 3). A spectrophotometer at a wavelength of 610 nm was used to measure the absorbance. The data were expressed as Evans blue (*μ*g)/tissue (g).

### 2.7. Brain Edema

Mice were sacrificed under anesthesia on day 3 following ICH. The brain sample was immediately collected and divided into two. The samples were weighed to access the data of wet weight and then dehydrated under constant high temperature for 1 day to obtain the data of dry weight. The brain content was calculated as wet and dry weight difference/wet weight∗100%.

### 2.8. Nissl's Staining

Animals were euthanized on day 3 after operation. The brain samples were harvested, frozen, cut, and stained with Nissl staining in sequence. The sections at different areas including the hippocampus (Hip), perihematomal (Per), and cortex (Cor) were photographed.

### 2.9. Terminal Deoxynucleotidyl Transferase-Mediated dUTP Nick 3′-End Labeling (TUNEL)

On day 3 after surgery, mice were transcardially perfused with 0.9% ice-cold saline and 4% paraformaldehyde (Sangon, Shanghai, China) under anesthesia, successively. Then, the corresponding brain samples were collected, fixed in 4% paraformaldehyde for another 1 d, and dehydrated by 25% sucrose for 3 d. The samples were embedded in paraffin and cut into pieces. A TUNEL kit (Beyotime Biotechnology, Haimen, Jiangsu, China) was employed to stain the paraffin pieces according to the manufacturer's protocols.

### 2.10. Quantitative Real-Time Polymerase Chain Reaction (RT-PCR)

Perihematomal brain tissues were harvested from each group at 3 days following surgery. Total RNA was extracted using Trizol Reagents (Invitrogen, Carlsbad, CA, USA) and verified by the spectrophotometric analysis (OD_260/280_). Primers used in the current study were synthesized commercially by Sangon Co. Ltd. (Shanghai, China) as follows: NLRP3: AGCCTTCCAGGATCCTCTTC (forward), CTTGGGCAGCAGTTTCTTTC (reverse); ASC: CTTAGAGACATGGGCTTACAGG (forward), CTCCAGGTCCATCACCAAGTAG (reverse); caspase-1: ACACGTCTTGCCCTCATTATCT (forward), TTTCACCTCTTTCACCATCTCC (reverse); IL-1*β*: TCATTGTGGCTGTGGAGAAG (forward), AGGCCACAGGTATTTTGTCG (reverse); IL-18: AAGAACAAGATCATTTCCTTTGAGGA (forward), GGAACACGTTTCTGAAAGAATATGAG (reverse); interleukin-6 (IL-6): AGTCCGGAGAGGAGACTTCA (forward), ATTTCCACGATTTCCCAGAG (reverse); monocyte chemotactic protein 1 (MCP-1): AGGTCCCTGTCATGCTTCTGG (forward), TGGTGATCCTCTTGTAGCTCTCC (reverse); tumor necrosis factor-*α* (TNF-*α*): CCCTCACACTCAGATCATCTTCT (forward), GCTACGACGTGGGCTACAG (reverse); and *β*-actin: GTGACGTTGACATCCGTAAAGA (forward), GCCGGACTCATCGTACTCC (reverse). The reverse transcription and subsequent RT-PCR analysis were performed with a Prime Script RT reagent kit and a SYBR Green kit (both from TaKaRa, Otsu, Japan), respectively. The SDS software (Applied Biosystems, Carlsbad, CA, USA) was employed to calculate the results by the *Δ*Ct method.

### 2.11. Western Blot Analysis

Perihematomal brain tissues were harvested on day 3 after operation. The total proteins, nuclear and cytoplasmic proteins, and mitochondrial proteins were extracted with the RIPA lysis buffer (Millipore, Bedford, MA, USA), Nuclear and Cytoplasmic Protein Extraction Kit (Beyotime Biotechnology, Haimen, Jiangsu, China), and Mitochondrial Protein Extraction Kit (BestBio, Shanghai, China), respectively. The quantitative proteins were loaded onto 10% sodium dodecyl sulfate-polyacrylamide gel by electrophoresis (SDS-PAGE) and wet-transferred to a 0.22 or 0.45 *μ*m PVDF membrane (Millipore, Temecula, CA, USA). At room temperature, the immunoblots were blocked by 5% nonfat milk for 1 h. Then, the immunoblots were probed with the target primary antibodies against PARP1 (1 : 5,000 dilution, catalog no. ab227244), Bcl-2 (1 : 1,000 dilution, catalog no. ab194583), NLRP3 (1 : 1,000 dilution, catalog no. ab4207), caspase-1 (1 : 1,000 dilution, catalog no. ab1872), IL-18 (1 : 400 dilution, catalog no. ab71495), NF-*κ*B (1 : 1,000 dilution, catalog no. ab19870), Nrf2 (1 : 1,000 dilution, catalog no. ab137550), OPTN (1 : 300 dilution, catalog no. ab23666), Beclin1 (1 : 2,000 dilution, catalog no. ab207612), Parkin (1 : 500 dilution, catalog no. ab77924), CoxIV (1 : 2,000 dilution, catalog no. Ab33985), and *β*-actin (1 : 5,000 dilution, catalog no. ab8227, all from Abcam, Cambridge, UK); Bax (1 : 1,000 dilution, catalog no. 2772), ASC (1 : 1,000 dilution, catalog no. 67824), IL-1*β* (1 : 1,000 dilution, catalog no. 52718), LC3 (1 : 1,000 dilution, catalog no. 4108), PINK1 (1 : 1,000 dilution, catalog no. 6946), and Histone H3 (1 : 1,000 dilution, catalog no. 4499, all from Cell Signaling Technology, Beverly, MA, USA) at 4°C overnight. After incubation with the corresponding secondary antibodies (1 : 5,000 dilution, Hua An, Hangzhou, Zhejiang, China) for another 1 h at room temperature, the ECL chemiluminescence (CL) reagent (Pierce, Rockford, IL, USA) was employed to detect the protein signals. The final results were quantified with the Quantity One software (Bio-Rad, Hercules, CA, USA).

### 2.12. Enzyme-Linked Immunosorbent Assay (ELISA)

The protein levels of IL-1*β*, IL-18, IL-6, MCP-1, and TNF-*α* in the perihematomal brain samples were detected by the IL-1*β* ELISA kit (Abcam, Cambridge, UK), IL-18 ELISA kit (MBL, Nagoya, Japan), IL-6 ELISA kit, MCP-1 ELISA kit, and TNF-*α* ELISA kit (all three from R&D Systems, Minneapolis, MN, USA) according to the manufacturer's instructions. A spectrophotometer was employed to measure the 450 nm absorbance value.

### 2.13. Caspase-1 Activity

The caspase-1 activity was detected as we previously described [10]. In brief, perihematomal brain samples were quick-freezed and fractured in a reaction buffer which contains 50 mM NaCl, 10% glycerol, 1 mM DTT, 1 mM EDTA, 1 mM bestatin, 1 mM pepstatin, 1 mM 4-(2-aminoethyl)benzenesulfonyl fluoride hydrochloride, and 50 mM HEPES (pH 7.4) by using the TissueLyser II kit (Qiagen, Valencia, CA, USA). After centrifugation and quantification, the protein concentration was adjusted to a final concentration of 2.5 mg/ml. The substrate of caspase-1, Ac-YVAD-p-nitroaniline (p-NA) (Enzo Life Sciences, Farmingdale, NY, USA) was further employed to detect the activity of caspase-1 colorimetrically in the clarified lysates. The data were calculated as (Δ[p − NA]/Δtime)/(total protein) and expressed as the relative changes relative to the controls finally.

### 2.14. Immunohistochemistry

Perihematomal brain samples were harvested on day 3 after operation, and the standard immunohistochemistry was performed. Briefly, the primary antibodies used were as follows: anti-PARP1 (1 : 400 dilution, catalog no. ab227244), NLRP3 (1 : 1,000 dilution, catalog no. ab4207), and caspase-1 (1 : 1,000 dilution, catalog no. ab1872, all from Abcam, Cambridge, UK). The data were determined by evaluating the staining intensity (five grades) as our previous study [16].

### 2.15. Luminol and Lucigenin Chemiluminescence (CL) Assays

Perihematomal brain samples were harvested on day 3 postoperation and fractured in the working buffer (20 mM HEPES in 0.5 M PBS, pH 7.2). Then, the luminol and lucigenin (both from Sigma-Aldrich, St. Louis, MO, USA) were added and adjusted to a final concentration of 0.2 mM. Changes in the curve area over 5 min were detected. The data were expressed as the relative light units (rlu)/mg brain tissues.

### 2.16. Malondialdehyde (MDA) Content and Total Superoxide Dismutase (SOD) Activity Assays

Perihematomal brain samples were collected on day 3 after operation and homogenized in the precooling saline. After centrifugation, the Tissue MDA Content Kit and Tissue Total SOD activity kit (Jianchen, Nanjing, Jiangsu, China) were employed to detect the MDA and SOD activity levels, respectively.

### 2.17. Mitochondrial ROS

Brain samples harvested from each group on day 3 after operation were dispersed to single-cell suspension using a sterile pipettor. The MitoSOX™ (5 mM, Thermo Fisher, Waltham, USA) reagent stock solution was diluted with HBSS/Ca/Mg buffer for the preparation of a final concentration of 5 *μ*M working solution. After washing with PBS twice, the cells were incubated in the 1 ml MitoSOX reagent working solution for 10 min at 37°C. A spectrophotometer at ex/em = 510/580 nm was employed to detect the absorbance.

### 2.18. Transmission Electron Microscope (TEM)

The animals were transcardially perfused with 4% paraformaldehyde and 2.5% glutaraldehyde in 0.1 M sodium cacodylate buffer (pH 7.4) under deep anesthesia prior to sacrifice on day 3 after operation. After perfusion, about 1 × 1 × 1 mm^3^ brain blocks were collected from the perihematomal regions of the animals. Afterward, the blocks were immersed in 2.5% glutaraldehyde for 2 h at 4°C. In subsequence, the samples were fixed with 1% OsO_4_ and then dehydrated in ethyl alcohol. Following infiltration with propylene oxide, the samples were embedded in Eponate and sectioned. Then, the sections were stained with 2% uranyl acetate and Reynolds' lead citrate. The TEM images were captured using a TEM (Philips, Amsterdam, The Netherlands).

### 2.19. Coimmunoprecipitation (Co-IP) Assay

The Flag or Myc-tagged proteins were immunoprecipitated as we previously described [20]. In brief, the overexpression plasmid of Nrf2, OPTN, and according empty vectors was purchased from Shanghai GenePharma Co., Ltd (Shanghai, China). According to the manufacturer's protocol, HEK-293T cells were transiently cotransfected with the Myc-Nrf2 and the Flag-OPTN vector or empty vector for 48 h. The transfected cells were collected and lysed in immunoprecipitation lysis buffer supplemented with protease inhibitor cocktail. Afterward, the cell lysates were incubated with anti-flag antibody-conjugated agarose beads or antibodies (1-2 *μ*g) for 4 h at 4°C, followed by another 1 h incubation with protein A-Sepharose beads (GE Healthcare, Waukesha, USA) if free antibody was used. Immunoprecipitates were washed, resolved by SDS-PAGE, and then immunoblotted with the appropriate antibodies.

### 2.20. Statistical Analysis

SPSS 16.0 (SPSS Inc., Chicago, IL, USA) was employed to analyze the results. Differences between two groups were compared with Student's *t*-test. Statistical comparisons were analyzed using one-way ANOVA followed by the Student-Newman-Keuls method. The difference of mice survival rate was detected by the Kaplan-Meier method followed by the log-rank test. The quantitative data were expressed as mean ± SD. *p* < 0.05 was considered to suggest a statistically significant difference.

## 3. Results and Discussion

### 3.1. Identification of DEPs and Bioinformatics via iTRAQ Quantification

**Figure 1:**
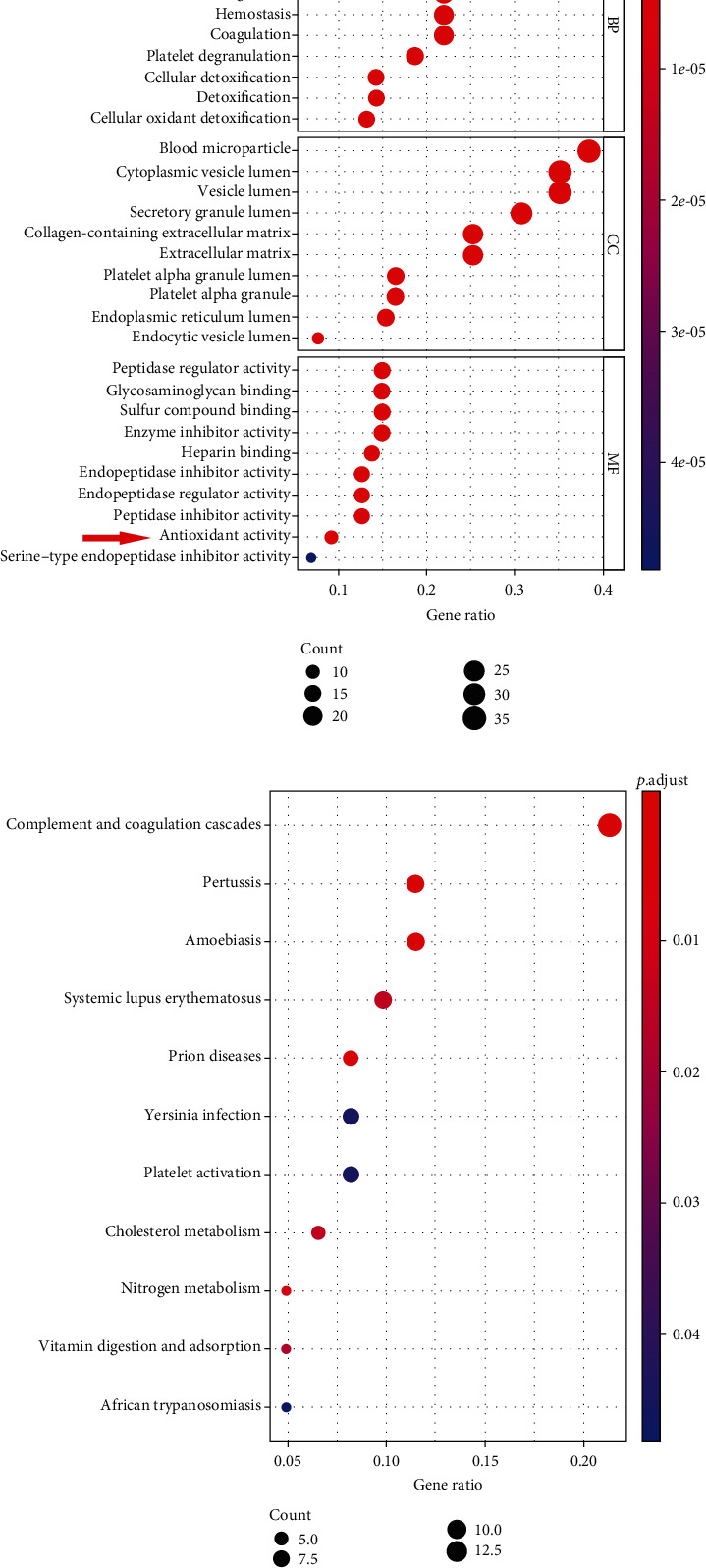
Identification of bioinformatics via iTRAQ quantification. (a) The GO function analysis; (b) the KEGG pathway analysis. *n* = 4.

Protein extracts from human perihematomal and normal brain tissues were collected for the iTRAQ analysis. In total, we found that there were 393 DEPs. GO enrichment analysis showed that these DEPs were mainly involved in the CC category, such as the blood microparticle, cytoplasmic vesicle lumen, and vesicle lumen. In particular, we noticed that the acute inflammatory response and antioxidant activity which were key pathophysiological responses of SBI following ICH accounted for a significant proportion in the BP and MF category, respectively (Figure 1(a)). For pathway analysis of these 393 DEPs, a total of 13 significant pathways were mapped by the KEGG database. The top 10 KEGG pathway analysis demonstrated that these DEPs mainly participated in the complement and coagulation cascades, pertussis, amoebiasis, etc. (Figure 1(b)).

### 3.2. Identification and Validation of the Targeted Proteins

**Figure 2:**
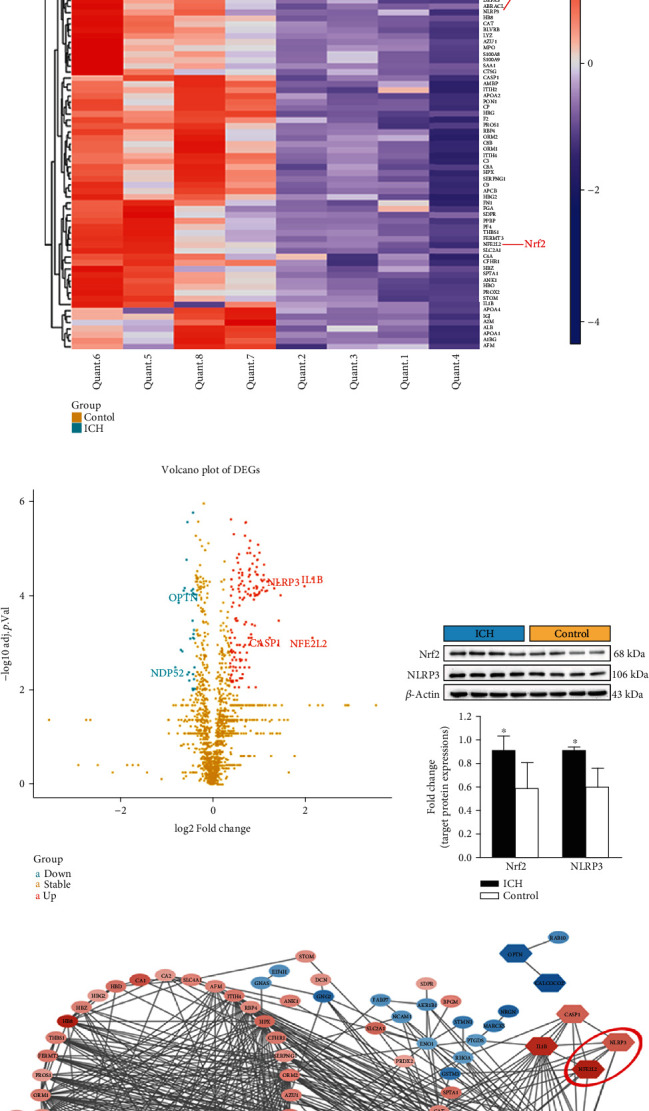
Identification and validation of the targeted proteins. (a) The heat map of DEPs; (b) the volcano plot of DEPs; (c) representative immunoblots and bar graph of densitometric analysis of Nrf2 and NLRP3 proteins from clinical specimens; (d) the functional PPI network. The values are presented as mean ± SD; *n* = 4; ^∗^*p* < 0.05 vs. control group.

To systematically identify abnormally DEPs potentially involved in the pathological progression of SBI following ICH, the heat map was generated to visualize the hierarchical cluster analysis. Among these abnormally expressed proteins, we found that both the key regulators of acute inflammatory response and antioxidant activity, Nrf2 and NLRP3 translations were remarkably upregulated in human perihematomal samples relative to normal brain samples (Figure 2(a)). Moreover, the volcano plot was sketched to clearly represent the protein abundance changes in human brain samples of ICH and control groups, respectively. The data showed that Nrf2, NLRP3, and NLRP3 inflammasome components caspase-1 were significantly upregulated proteins (Figure 2(b)). To verify the target iTRAQ quantification results, we performed Western blot analysis to detect the target protein expressions of Nrf2 and NLRP3 in human perihematomal and normal brain tissues. Consistent with the iTRAQ profile data, Western blot results confirmed that Nrf2 and NLRP3 protein expressions were higher in the human perihematomal brain tissues compared to that in the normal brain tissues (Figure 2(c)). Additionally, we examined Nrf2 and NLRP3 mRNA expressions in human perihematomal and normal brain samples by RT-PCR. Compared with the human normal brain tissues, the mRNA levels of Nrf2 and NLRP3 were also higher in human perihematomal brain tissues (*p* < 0.01; Figure S1). To further investigate the possible interaction of these DEPs, we performed PPI analysis to analyze the above-mentioned DEPs whose fold change > 2.0 and *p* < 0.05. In particular, we found that Nrf2 and NLRP3 were located at the hub positions of the functional PPI network and interconnected closely (Figure 2(d)).

### 3.3. Nrf2 Deficiency Decreased Survival Rate, Enlarged Hemorrhagic Lesion Volumes, and Aggravated Neurological Deficits after ICH

**Figure 3:**
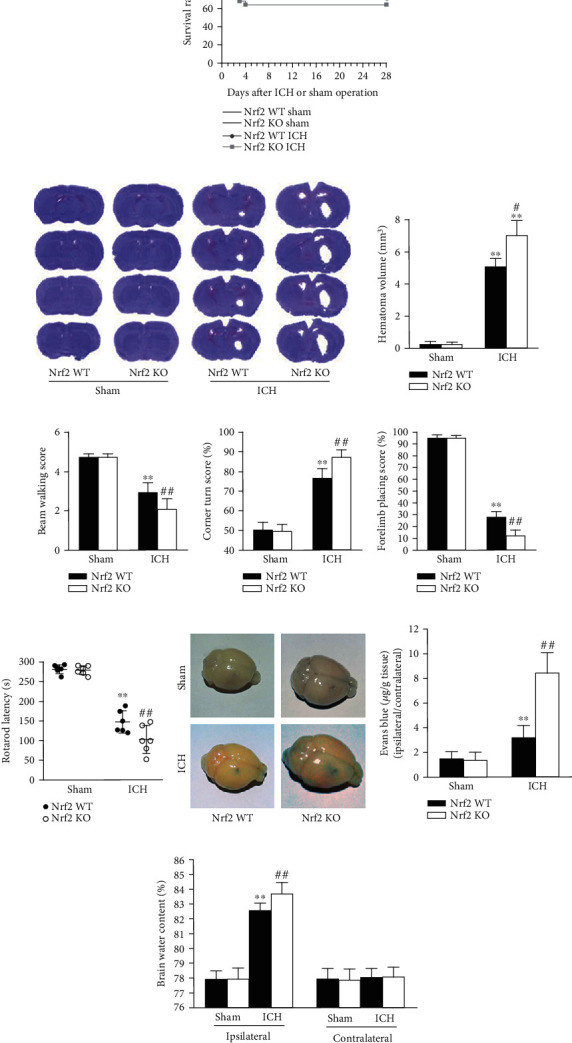
Effects of Nrf2 deficiency on the SBI after ICH in mice. (a) Effect of Nrf2 on the survival rate after ICH. The survival rates were recorded during the following 28 days after operation in the Nrf2 WT sham (*n* = 13), Nrf2 KO sham (*n* = 13), Nrf2 WT ICH (*n* = 18), and Nrf2 KO ICH (*n* = 25) groups, respectively. (b) Effect of Nrf2 deficiency on the hemorrhagic lesion volumes after ICH. (c–f) Effect of Nrf2 on the neurological deficits after ICH. In the beam walking (c), corner turn (d), forelimb placing (e), and Rotarod (f) tests, the beam walking score, percent of left turns, percent of left paw placement, and latency to fall were scored in each group on day 3 after surgery, respectively. *n* = 6. (g) Effect of Nrf2 on the BBB integrity after ICH. (h) Effect of Nrf2 on the brain edema after ICH. *n* = 3. ^∗^*p* < 0.05 and ^∗∗^*p* < 0.01 vs. Nrf2 WT sham group; ^#^*p* < 0.05 and ^##^*p* < 0.01 vs. Nrf2 WT ICH group.

To further explore the effects of target protein Nrf2 on the initiation and development of SBI following ICH, Nrf2 WT and Nrf2 KO mice were employed to conduct *in vivo* experiments. Firstly, we used the Kaplan-Meier method to observe the effect of Nrf2 on mouse survival post-ICH. The number of mice that died of natural causes in different groups was recorded during the following 28 days after ICH induction or sham operation. As shown in Figure 3(a), the survival rate was 100% in both the Nrf2 WT and Nrf2 KO sham groups. There was no statistical difference between the Nrf2 WT mice with or without ICH operation (*p* > 0.05). However, the survival rate was significantly decreased in the Nrf2 KO ICH group than in the Nrf2 KO sham group (*p* < 0.05). Although there was no statistical difference, the Nrf2 KO mice had a relatively lower survival rate compared with the Nrf2 WT group after ICH (*p* > 0.05).

To evaluate the effect of Nrf2 on the brain injury after ICH directly, we secondly detected the hematoma volumes on day 3 after ICH induction. As shown in Figure 3(b), ICH operation induced markedly bigger hematoma volumes than sham surgery (*p* < 0.01). Moreover, ICH-induced hemorrhagic lesion volumes were significantly bigger in the Nrf2 KO mice than in the Nrf2 WT mice (*p* < 0.01).

Thirdly, we performed the neurofunctional tests to further investigate the effect of Nrf2 on SBI after ICH. As shown in Figures 3(c)–3(f), ICH surgery induced significantly neurofunctional deficits according to the beam walking, corner turn, forelimb placing, and Rotarod scores relative to sham operation in both the Nrf2 WT and Nrf2 KO mice (all *p* < 0.01). Compared with the Nrf2 WT ICH mice, the Nrf2 KO ICH mice had remarkably worse neurologic scores in all the beam walking, corner turn, forelimb placing, and Rotarod tests on day 3 (all *p* < 0.01; Figures 3(c)–3(f)).

### 3.4. Nrf2 Deficiency Exacerbated BBB Disruption and Brain Edema after ICH

Afterward, we test the effect of Nrf2 on the BBB integrity and brain edema on day 3 after ICH. As shown in Figure 3(g), low Evans blue extravasation was detected in both the Nrf2 WT and Nrf2 KO sham mice, while ICH induction caused a significant increase in the level of dye extravasation compared to the sham groups (*p* < 0.01). In addition, the Nrf2 KO mice had an even higher level of Evans blue leakage compared to the Nrf2 WT mice after ICH induction (*p* < 0.01).

As for the brain edema, ICH induced a significant increase in the brain water contents relative to the sham groups in both the Nrf2 WT and Nrf2 KO mice on day 3 in the ipsilateral hemispheres, respectively (both *p* < 0.01; Figure 3(h)). Moreover, ICH-induced brain edema was significantly aggravated in the Nrf2 KO mice than in the Nrf2 WT mice (*p* < 0.01), while there were no significant differences among any of the groups in the contralateral hemispheres (all *p* > 0.05).

### 3.5. Nrf2 Deficiency Affected Neuronal Survival of Remote Areas after ICH

**Figure 4:**
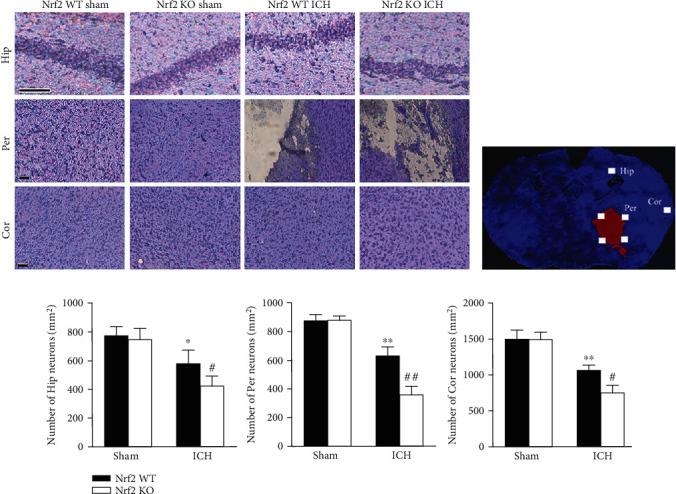
Effect of Nrf2 on the neuronal survival of remote areas after ICH. (a) Representative photomicrography of Nissl-stained brain sections from each group. Scale bars = 100 *μ*m. (b) The schematic diagram explaining the imaging protocol for the quantification of Nissl-staining neurons. (c) Quantification analysis of the number of Nissl-positive neurons in each group, respectively. The values are presented as mean ± SD; *n* = 3; ^∗^*p* < 0.05 and ^∗∗^*p* < 0.01 vs. Nrf2 WT sham group; ^#^*p* < 0.05 and ^##^*p* < 0.01 vs. Nrf2 WT ICH group.

To assess whether Nrf2 affected neuronal death in regions remote from the hematoma, the Hip, Per, and Cor brain tissues were collected for Nissl staining. Compared to the sham groups, ICH mice from both the Nrf2 WT and Nrf2 KO groups had significantly less Nissl-positive neurons in all the Hip, Per, and Cor areas (*p* < 0.01, Figure 4(a)). Moreover, the Nrf2 KO ICH mice had significantly even less number of Nissl-positive neurons than the Nrf2 WT ICH mice in all the Hip, Per, and Cor areas (*p* < 0.01; Figure 4(a)).

**Figure 5:**
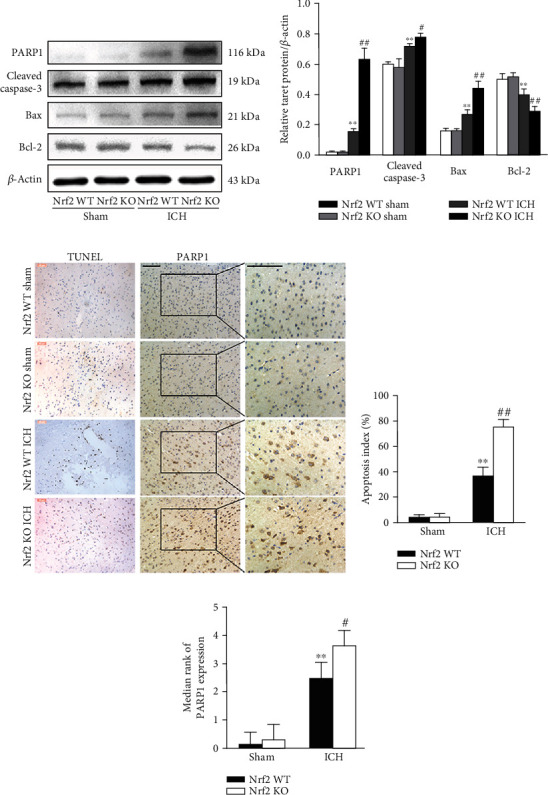
Effect of Nrf2 on the perihematomal neuronal apoptosis after ICH. (a) Representative immunoblots of PARP1, cleaved caspase-1, Bax, and Bcl-2 proteins from each group. (b) The bar graph of densitometric analysis shows the protein levels of PARP1, cleaved caspase-1, Bax, and Bcl-2 in each group. (c) Representative photomicrography of TUNEL- and PARP1-stained brain sections from each group. Scale bars = 50 and 100 *μ*m, respectively. (d) Quantification analysis of the percentage of TUNEL-positive cells in each group, respectively. (e) Quantification analysis indicates the median ranks of PARP1 expression in each group. The values are presented as mean ± SD; *n* = 3‐6; ^∗∗^*p* < 0.01 vs. Nrf2 WT sham group; ^#^*p* < 0.05 and ^##^*p* < 0.01 vs. Nrf2 WT ICH group.

Furthermore, we conducted TUNEL assay and Western blot analysis to detect the effect of Nrf2 on the perihematomal neuronal apoptosis. As shown in Figure 5(a), Western blot analysis showed that ICH-induced upregulation of PARP1, cleaved caspase-3, and Bax protein expressions was significantly enhanced by Nrf2 KO (*p* < 0.05). On the contrary, ICH-induced downregulation of Bcl-2 protein expression was further decreased by Nrf2 KO (*p* < 0.05). Accordingly, TUNEL and immunohistochemistry assays showed that few TUNEL-positive and PARP1-positive cells were observed in the perihematomal areas from both the Nrf2 WT and Nrf2 KO sham mice. Compared to the sham groups, ICH mice from both the Nrf2 WT and Nrf2 KO groups had significantly more TUNEL-positive and PARP1-positive cells (*p* < 0.05, Figures 5(c) and 5(d)). Additionally, the Nrf2 KO ICH mice had a markedly higher apoptosis index and median rank of PARP1 expression than the Nrf2 WT ICH mice (*p* < 0.01; Figures 5(c) and 5(d)).

### 3.6. Nrf2 Deficiency Impaired the Endogenous Antioxidant Capacity after ICH

**Figure 6:**
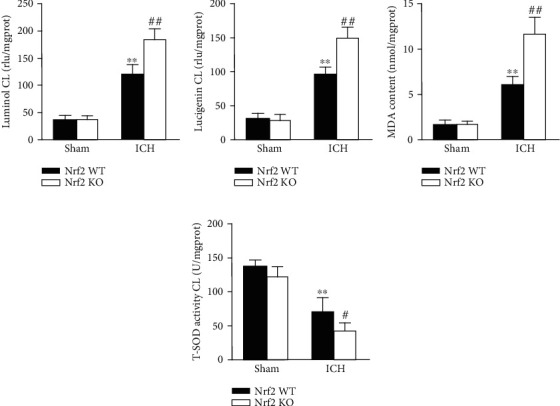
Effect of Nrf2 on the ROS generation after ICH. The levels of luminol CL (a), lucigenin CL (b), MDA content (c), and total SOD activity (d) in each group at 3 days after operation. The values are presented as mean ± SD; *n* = 6; ^∗∗^*p* < 0.01 vs. Nrf2 WT sham group; ^#^*p* < 0.05 and ^##^*p* < 0.01 vs. Nrf2 WT ICH group.

As known, Nrf2 is the key regulator of the cellular endogenous antioxidant system. To examine the effect of Nrf2 deficiency on the oxidative stress after ICH, we conducted the CL assays of luminol and lucigenin, MDA content, and total SOD activity assays. As shown in Figures 6(a)–6(c), the levels of luminal CL, lucigenin CL, and MDA content were remarkedly increased in the Nrf2 KO mice compared to the Nrf2 WT mice after ICH (all *p* < 0.01). Inversely, the level of T-SOD activity was significantly decreased in the Nrf2 KO mice relative to the Nrf2 WT mice following ICH (Figure 6(d); *p* < 0.05).

### 3.7. Nrf2 Deficiency Enhanced NLRP3 Inflammasome Activation and Subsequent Neuroinflammation after ICH

**Figure 7:**
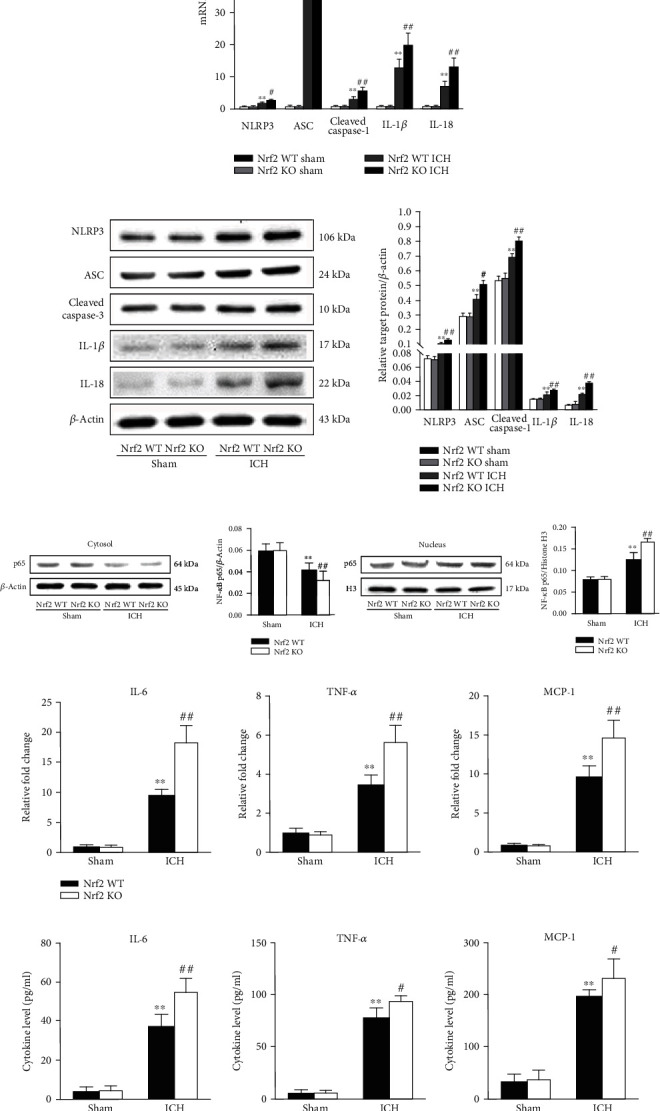
Effect of Nrf2 on the NLRP3 inflammasome activation and neuroinflammation after ICH. (a) The fold changes of NLRP3, ASC, caspase-1, IL-1*β*, and IL-18 mRNA levels in different groups. (b) Representative immunoblots and bar graph of densitometric analysis of NLRP3, ASC, cleaved caspase-1, IL-1*β*, and IL-18 proteins from each group. (c) Representative immunoblots and bar graph of densitometric analysis of cytosol and nucleus NF-*κ*B proteins from each group. (d) The fold changes of IL-6, MCP-1, and TNF-*α* mRNA levels in different groups. (e) The protein levels of IL-6, MCP-1, and TNF-*α* in each group. The values are presented as mean ± SD; *n* = 6; ^∗^*p* < 0.05 and ^∗∗^*p* < 0.01 vs. Nrf2 WT sham group; ^#^*p* < 0.05 and ^##^*p* < 0.01 vs. Nrf2 WT ICH group.

To test whether Nrf2 could inhibit NLRP3 inflammasome activation after ICH, the RT-PCR, Western blot analysis, ELISA, and caspase-1 activity assays were performed. As shown in Figure 7(a), the Nrf2 KO mice showed higher mRNA levels of NLRP3, ASC, caspase-1, IL-1*β*, and IL-18 compared to the Nrf2 WT mice after ICH induction on day 3 (all *p* < 0.01). Consistent with the mRNA results, the Nrf2 KO mice also had higher protein levels of NLRP3, ASC, cleaved caspase-1, IL-1*β*, and IL-18 relative to the Nrf2 WT mice after ICH (all *p* < 0.01; Figure 7(b)). Moreover, we performed the ELISA assays to further detect the changes in IL-1*β* and IL-18 releases in each group. Accordingly, the results showed that ICH-induced upregulations of IL-1*β* and IL-18 release were even higher in the Nrf2 KO mice when compared with the Nrf2 WT mice (both *p* < 0.01; Figure S2). As the caspase-1 activity directly determines the maturation and release of IL-1*β* and IL-18, we further performed the caspase-1 activity assay. As expected, the caspase-1 activity was more active in the Nrf2 KO ICH group than in the Nrf2 WT ICH group (*p* < 0.01; Figure S3).

As NLRP3 inflammasome provides a platform for the initiation and aggravation of neuroinflammatory cascade reaction after ICH, to determine the effect of Nrf2 on the subsequent neuroinflammation after ICH, we firstly detected the protein level of NF-*κ*B in each group. Firstly, we measured the changes in the NF-*κ*B activation in each group. As shown in Figure 7(c), ICH-induced downregulation of the cytoplasmic NF-*κ*B p65 protein level was further decreased in the Nrf2 KO ICH group than in the Nrf2 WT ICH group (*p* < 0.01). Contrarily, ICH-induced upregulation of the nuclear NF-*κ*B p65 protein level was further increased in the Nrf2 KO mice than in the Nrf2 WT mice (*p* < 0.01). Secondly, we further employed RT-PCR and ELISA assays to test the mRNA and protein expression changes of proinflammatory cytokines. As shown in Figure 7(d), the mRNA levels of IL-6, MCP-1, and TNF-*α* were significantly upregulated in the Nrf2 KO ICH group when compared with the Nrf2 WT ICH group (all *p* < 0.01). As anticipated, the protein levels of IL-6, MCP-1, and TNF-*α* were also significantly increased in the Nrf2 KO mice relative to the Nrf2 WT mice after ICH induction on day 3, respectively (all *p* < 0.01; Figure 7(e)).

### 3.8. Nrf2 Deficiency Aggravated Mitochondrial Dysfunction and Impaired Mitophagy after ICH

**Figure 8:**
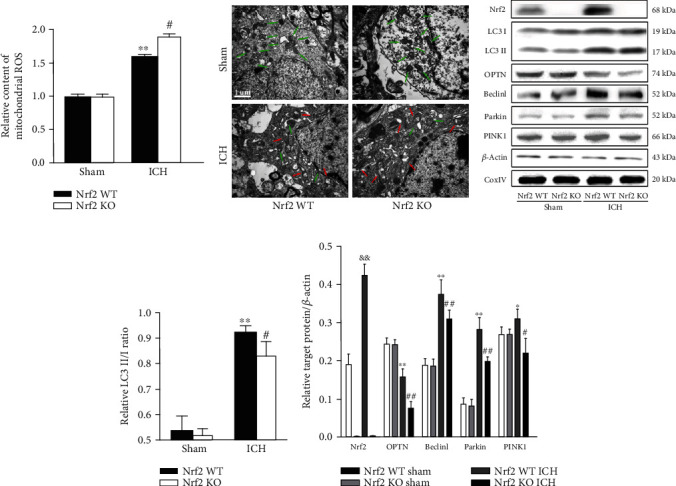
Effect of Nrf2 on the mitochondrial function and mitophagy after ICH. (a) Effect of Nrf2 on the mtROS in each group. (b) Effect of Nrf2 on the mitochondrial ultrastructure in each group. (c) Representative immunoblots of Nrf2, LC3 II/I, OPTN, Beclin1, Parkin, and PINK1 proteins from each group. The bar graph of densitometric analysis shows the protein levels of LC3 II/I (d), Nrf2, OPTN, Beclin1, Parkin, and PINK1 (e) in each group. The values are presented as mean ± SD; *n* = 3‐6; ^∗^*p* < 0.05 and ^∗∗^*p* < 0.01 vs. Nrf2 WT sham group; ^#^*p* < 0.05 and ^##^*p* < 0.01 vs. Nrf2 WT ICH group; ^&&^*p* < 0.01 vs. Nrf2 WT sham group.

Mitochondrial dysfunction is closely involved in the activation of NLRP3 inflammasome. To assess the changes in the mitochondrial function in different groups, we performed the MitoSOX test and TEM observation. As shown in Figure 8(a), ICH-induced upregulation of mtROS was further markedly increased in the Nrf2 KO mice when compared with the Nrf2 WT mice (*p* < 0.05). Furthermore, we observed the ultrastructural changes of mitochondria in each group using a TEM. As shown in Figure 8(b), brain blocks from the Nrf2 WT and Nrf2 KO sham mice showed healthy and intact mitochondrial structure. Nevertheless, ICH induction caused obviously mitochondrial ultrastructural damages, including swelling, dark mitochondrial matrix, and irregularly arranged mitochondrial cristae. Compared with the Nrf2 WT ICH group, even more damaged mitochondria were observed in the Nrf2 KO ICH group.

Cells remove the damaged mitochondria mainly through mitophagy. To evaluate the effect of Nrf2 on mitophagy in each group, we performed the Western blot analysis. As shown in Figure 8(c), the mitochondrial protein levels of LC3 II/I, OPTN, Beclin1, Parkin, and PINK1 were markedly decreased in the Nrf2 KO mice relative to the Nrf2 WT mice after ICH induction (all *p* < 0.05).

### 3.9. OPTN-Mediated Mitophagy Suppressed NLRP3 Inflammasome and SBI after ICH

**Figure 9:**
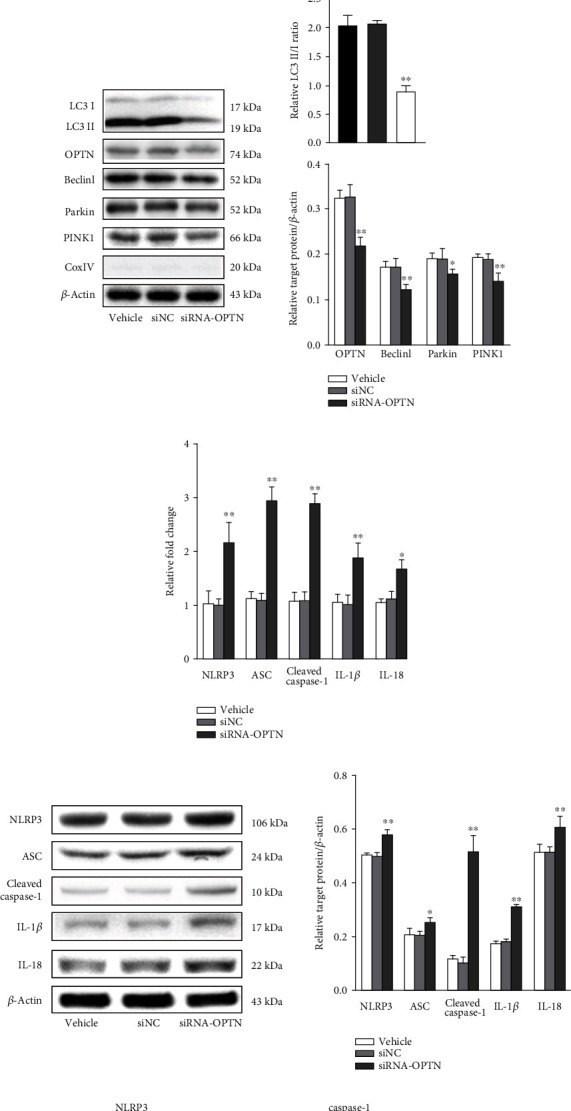
Effect of OPTN on the Nrf2-mediated mitophagy and neuroprotection after ICH. (a) GEPIA predicting the association between Nrf2 and OPTN. (b) Representative Co-IP immunoblots of Nrf2 and OPTN. (c) Effect of OPTN on the mtROS in each group. (d) Representative immunoblots and bar graph of densitometric analysis of mitochondrial LC3 II/I, OPTN, Beclin1, Parkin, and PINK1 proteins from each group. (e) Representative immunoblots and bar graph of densitometric analysis of nuclear and cytoplasmic LC3 II/I, OPTN, Beclin1, Parkin, and PINK1 proteins from each group. (f) The fold changes of NLRP3, ASC, cleaved caspase-1, IL-1*β*, and IL-18 mRNA levels in different groups. (g) Representative immunoblots and bar graph of densitometric analysis of NLRP3, ASC, cleaved caspase-1, IL-1*β*, and IL-18 proteins from each group. (h) Representative photomicrography of NLRP3- and caspase-1-stained brain sections from each group. Scale bars = 100 *μ*m, respectively. (i) Effect of OPTN on the neurological deficits after ICH. The values are presented as mean ± SD; *n* = 3‐6; ^∗^*p* < 0.05 and ^∗∗^*p* < 0.01 vs. vehicle group.

The above-mentioned proteomics bioinformatics analyzed by iTRAQ quantification prompted that OPTN may play an important role in Nrf2-mediated mitophagy. Firstly, GEPIA analysis based on TCGA datasets was employed to predict the association between Nrf2 and OPTN. As shown in Figure 9(a), there is a close correlation between them. Secondly, Co-IP analysis further confirmed that Nrf2 can interact with OPTN (Figure 9(b)). Then, the siRNA technique was employed to further investigate the role of OPTN in mitophagy after ICH. As shown in Figure 9(c), ICH-induced upregulation of mtROS was significantly decreased in the siRNA-OPTN mice when compared with the si-NC mice (*p* < 0.05). Both the mitochondrial and nuclear and cytoplasmic protein levels of LC3 II/I, OPTN, Beclin1, Parkin, and PINK1 were significantly decreased after OPTN siRNA administration. However, both the mRNA and protein levels of NLRP3 inflammasome were increased after OPTN siRNA administration. Furthermore, immunostains for NLRP3 and caspase-1 were significantly more evident in the siRNA-OPTN group than in the si-NC group. In addition, ICH-induced neurofunctional deficits were remarkably improved after OPTN siRNA injection (*p* < 0.05).

## 4. Discussion

Employing iTRAQ quantification technology and Nrf2 KO mice, the current study suggested that Nrf2/OPTN-mediated mitophagy confers neuroprotective effects against neuroinflammatory insults via inhibiting the NLRP3 inflammasome activation after ICH, thereby alleviating SBI. To be specific, our data revealed that (i) clinical iTRAQ results revealed that the acute inflammatory response and antioxidant activity and their targeted DEPs, Nrf2 and NLRP3, were closely involved in the pathological responses of SBI following ICH; (ii) Nrf2 deficiency exacerbated SBI after ICH, which represented with decreased survival ratio, worsen neurofunctional deficits, intensified brain edema, impaired BBB integrity, and increased neuronal death after ICH; (iii) Nrf2 deficiency aggravated NLRP3 inflammasome activation and subsequent neuroinflammation post-ICH; (iv) Nrf2 deficiency enhanced ROS generation after ICH; (v) Nrf2 can interact with OPTN and induced OPTN-mediated mitophagy following ICH.

At present, most research schemes focus on the role of a single gene or protein, which cannot effectively reflect the molecular functional network of SBI after ICH. Given that ICH is a very complex pathophysiological process, which involves multigene, multipathway, and multifunctional protein-protein network interaction, avoiding the obvious limitations of traditional research methods, such as long research cycle, unfocused research direction, low success rate, and difficult translation in clinical practice, etc., has become an urgent problem to be solved. Systemic proteomics is an important approach to investigate the intricate, interrelated, and multifactorial nature of molecular interactions to various diseases. Compared with the traditional experimental approaches, advances in high-throughput proteomics analysis can enable researchers to identify the total DEP profiles and abnormal pathological process of SBI after ICH to explore the potentially molecular and pathogenic mechanisms on the macro level. Particularly, further according experiments can be performed to verify the targeted data-derived research hypothesis [21–24]. However, no ICH proteomics studies have been published so far. Herein, we for the first time employed iTRAQ coupled with nano-LC-MS/MS technology to identify the DEPs and pathophysiological responses in clinical specimens. The results showed that of the DEPs after ICH, regulations of blood microparticle and complement and coagulation cascades were the most importantly pathophysiological process and pathway, respectively, which were also documented in terms of vascular injury and hemorrhage [25, 26]. It is worth noting that another two important processes, the acute inflammatory response and antioxidant activity, also occupied prominent positions in the GO category, which have been reported to be two key pathophysiological processes to brain injury after ICH [5]. According to the heat map of DEPs, the central regulators of the acute inflammatory response and antioxidant activity, NLRP3 and Nrf2, were significantly upregulated in the ICH patients, indicating that Nrf2 and NLRP3 inflammasome were closely involved in the development of SBI after ICH.

Notably, our prior studies have proven that Nrf2 was upregulated in kinds of ICH animal models and could counteract SBI as an adaptive response [8, 9]. In the later series of studies, we also found that exogenous administration of Nrf2 activator, such as ghrelin, could significantly attenuate SBI after ICH [10]. To further directly confirm the neuroprotective effect of Nrf2, we employed Nrf2 KO mice to establish ICH murine models. In addition to the elevated hemorrhage volume, leukocyte infiltration, and oxidative stress which were previously reported by Wang et al. [11], we also found that Nrf2 deficiency decreased survival ratio and increased neurofunctional deficits, brain edema, BBB disruption, and neuronal death after ICH. As for the NLRP3 inflammasome, both previous studies from us [16] and others [27–29] have proven that the NLRP3 inflammasome was time-dependently activated and amplified neuroinflammation after ICH. In our subsequent studies, we found that interference with the NLRP3 inflammasome by exogenous drugs such as cordycepin [16] and ghrelin [10] effectively suppressed neuroinflammation and SBI following ICH. Systematically, these data again confirmed that Nrf2 and NLRP3 inflammasome could be potential targets for SBI alleviation after ICH.

More and more evidence indicates that there is a crosstalk between the Nrf2 and NLRP3 inflammasome. Indeed, the crosstalk between them is pathophysiologically important for kinds of diseases at different levels [30]. However, whether Nrf2 supports or suppresses the activation of NLRP3 inflammasome remains controversial. In an atherosclerosis mouse model, Freigang et al. revealed that Nrf2 was an essential positive regulator of NLRP3 inflammasome activation and subsequent IL-1*β*-mediated vascular inflammation, thus exacerbating atherosclerosis [31]. Similarly, another two inflammasome-related models of chronic kidney disease and sepsis-associated encephalopathy also demonstrated that Nrf2 deficiency decreased the activation of NLRP3 inflammasome and the release of its downstream productions IL-1*β* and IL-18, indicating that NLRP3 inflammasome activation depends on Nrf2 [32, 33]. Later, it was shown that Nrf2 is required for the activation of NLRP3 inflammasome in several murine and human immune cells, including the bone marrow-derived macrophages, keratinocytes, and THP-1 cells [34–36]. On the other hand, Nrf2 is generally considered to have an inhibitory effect of NLRP3 inflammasome activation. For example, Chu et al. found that NLRP3 inflammasome was significantly more activated in Nrf2 KO than WT mice in a subchronic PM2.5 exposure animal model [37]. Moreover, a growing body of evidence suggests that various Nrf2 activators, such as melatonin [38], isoliquiritigenin [39], cardamonin [40], dihydromyricetin [41], and DI-3-butylphthalide [42] could inhibit NLRP3 inflammasome activation in different kinds of inflammation-related diseases. The contradictory phenomenon is likely related to the different *in vitro* and *in vivo* disease models considered. Interestingly, our prior study revealed that ghrelin, a potent activator of Nrf2, has been shown to be capable of inhibiting NLRP3 inflammasome activation after ICH [10]. However, from these studies, it cannot be concluded that the NLRP3 inflammasome inhibitory effect of these compounds depends on Nrf2 comprehensively. In addition, it should be pointed out that the specific relationship between Nrf2 and NLRP3 inflammasome remains unknown in ICH. In the current study, the functional PPI network firstly suggested that Nrf2 and NLRP3 inflammasome are interconnected to each other after ICH, suggesting that the crosstalk between them is pathophysiologically important for SBI. To further test their interconnection in a consistent or opposite manner, we then applied the Nrf2 KO ICH murine models. As expected, Nrf2 deficiency significantly enhanced the activation of NLRP3 inflammasome and subsequent neuroinflammation after ICH, suggesting that Nrf2 can inhibit NLRP3 inflammasome activation after ICH.

So far, the molecular mechanisms underlying how Nrf2 inhibits NLRP3 inflammasome activation have not been clearly elucidated. Moreover, it is still a matter of debate which molecular mechanisms govern the activation of NLRP3 inflammasome [43]. Obviously, it is unlikely that different and structurally diverse molecules activating NLRP3 inflammasome in all kinds of cases are mediated by direct interaction with NLRP3 [30, 44], while ROS was centrally proposed as the common signal for NLRP3 inflammasome activation since almost all NLRP3 stimuli can induce ROS generation [45, 46]. ROS can be generated by different sources. Due to the area of the mitochondrial inner membrane and the enzyme activity of respiratory chain complex are much bigger than the sum of all other membrane areas and related enzyme activities in the cell, mitochondrial dysfunction-induced abnormal mitochondrial respiratory chain is the most important source of ROS. Besides, mitochondrial dysfunction-induced abnormal OXPHOS activity and mtDNA damage also lead to the activation of NLRP3 inflammasome [17]. Thus, mitochondrial dysfunction is the upstream core event that leads to the activation of NLRP3 inflammasome, which has been proven in several central nervous system diseases, such as Parkinson's disease [47, 48], postoperative cognitive dysfunction [49], cerebral ischemia-reperfusion injury [50, 51], and Alzheimer's disease [52], while it is not clear whether this activation mechanism also exists in ICH. Prior studies have shown that the mitochondrial respiratory chain and mitochondrial membrane potential were abnormal in the brain tissue surrounding hematoma and closely associated with SBI after ICH [53, 54]. In order to observe ICH-induced mitochondrial changes more directly, we employed TEM in the present study. Intuitively, we found that the number of dysfunctional mitochondria with swelling, cristae disappearance, and vacuolation increased significantly after ICH induction. Accordingly, mtROS generation was also elevated post-ICH. Thus, removing the damaged mitochondria in time is a crucial means of inhibiting NLRP3 inflammasome activation after ICH, thereby alleviating SBI.

Cells mainly take advantage of mitophagy to remove dysfunctional mitochondria to control mitochondrial quality. Although the effect of autophagy on promoting or inhibiting cell death is still controversial in ICH, mitophagy as an organelle-specific autophagy can remove the damaged mitochondria to maintain cellular homeostasis [55]. More recent studies have shown that Nrf2 can play a cellular protective role by inducing mitophagy. For example, Nrf2 can induce its downstream target gene p62/SQSTM1 expression, which mediates the degradation of ubiquitination substrates through the autophagy pathway [56]. Moreover, Zhang et al. found that Nrf2 can promote the expression of mitophagy receptor PHB2 and then induce mitophagy to alleviate brain injury after SAH [57]. Furthermore, Nrf2 has also been proven to induce mitophagy by promoting NIX and BNIP3 expressions in glioma and myocardial ischemia, respectively [58, 59]. However, whether Nrf2 can regulate mitophagy in ICH, and if so, what kind of molecular mechanism it is. Using the Nrf2 KO mouse ICH models, we found that Nrf2 deficiency markedly decreased mitophagy level after ICH, indicating that Nrf2 could induce mitophagy, thus inhibiting dysfunctional mitochondrial-induced NLRP3 inflammasome activation after ICH. To further explore the underlying mechanisms, we again analyzed the iTRAQ results from the clinical specimens and found that OPTN may play a crucial role in Nrf2-induced mitophagy after ICH. As a multifunctional mitophagy receptor, OPTN can recruit the autophagy factors ULK1, DFCP1, and WIPI1 to focal spots proximal to mitochondria, then initiate the formation of mitophagosome by binding to LC3 [19]. Thus, we proposed the hypothesis that Nrf2 promotes mitophagy via modulating OPTN expression after ICH. To prove the hypothesis, we accomplished four ends. Firstly, employing Nrf2 KO mouse ICH models, we found that Nrf2 deficiency decreased OPTN expression. Secondly, we employed the GEPIA software to predict the association between Nrf2 and OPTN. The data revealed that there is a positive correlation between them. Thirdly, we performed the Co-IP analysis to further confirm that Nrf2 can interact with OPTN. Finally, interference with OPTN by siRNA technology markedly decreased the mitophagy level and increased NLRP3 inflammasome activation after ICH. It should be noted that OPTN could confer neuroprotection even though its whole protein level declined after ICH onset. Meanwhile, Nrf2 elevated with feedback to maintain its downstream OPTN-mediated mitophagy via inhibiting OPTN down-regulation as a compensatory reaction. Moreover, OPTN expression declined much more in the ICH mice than that in the WT mice when the Nrf2 gene was KO. As for the reason why OPTN declined after ICH, it may be due to OPTN which may also be regulated by other genes, which should be further investigated. Taken together, these data confirmed the hypothesis and supported Nrf2/OPTN-mediated mitophagy as targeted treatment to suppress NLRP3 inflammasome activation and subsequent neuroinflammation to alleviate SBI after ICH.

Although the current study provides proof of concept and elucidates the neuroprotective effect and new molecular mechanisms of Nrf2 in an ICH murine model, there are some potential limitations deserving attention in our experiments. Firstly, according to the PPI functional network, Nrf2 was located at the hub position, indicating Nrf2 plays a core role in the SBI after ICH. Prior studies from us and others mainly focused on the antioxidative and anti-inflammatory neuroprotective effects of Nrf2; however, more current studies have revealed that Nrf2 may also confer cerebroprotective effects via mitophagy in a SAH model [57]. Whether this effect also exists in ICH, and through what downstream molecular mechanisms have yet to be determined, in the current study, we employed Nrf2 KO mice and found that Nrf2 indeed modulated mitophagy level following ICH. To further investigate the molecular mechanisms, we analyzed the DEPs and found that the expression of pivotal mitophagy receptor, OPTN was significantly changed. Thus, we selected Nrf2/OPTN as the research object on the basis of iTRAQ-based quantitative proteomics. Additionally, OPTN may be only partially responsible for Nrf2-induced mitophagy after ICH, which is chosen based on the bioinformatics sequencing results. The other related mitophagy receptors such as BNIP3, NIX, and PHB3 need to be further verified. Secondly, OPTN overexpression rather than OPTN siRNA techniques should be employed to better observe its modulatory effects on mitophagy and neuroinflammation following ICH. Thirdly, although the crosstalk and inverse correlation between Nrf2 and NLRP3 inflammasome become evident, the mitophagy may be only partial mechanism. Other Nrf2-mediated responsible NLRP3 inflammasome inhibiting signals such as endoplasmic reticulum stress [60], and pannexin 1 channels [61] need to be further evaluated.

## 5. Conclusions

In conclusion, the current study revealed that Nrf2 could attenuate SBI following ICH by improving survival rate, attenuating hemorrhage volume, increasing neurological functional outcomes, alleviating BBB disruption and brain edema, and reducing perihematomal cell death. Mechanistic studies for the first time indicated that Nrf2 confers cerebroprotective effects by not only suppressing oxidative stress but also inhibiting NLRP3 inflammasome activation via inducing OPTN-mediated mitophagy. Notably, more and more inducers of Nrf2 such as Oltipraz, BG-12, and hydralazine were approved in succession by FDA and widely used in clinic. With the increased experience of Nrf2 activator administration in human beings [62], Nrf2 possesses the potential to be a useful molecular target in the clinical treatment of ICH.

## Data Availability

The data used to support the findings of this study are available from the corresponding author upon request.

## Supplementary Materials

Supplementary Materials Figure S1: the fold changes of Nrf2 and NLRP3 in ICH and control groups. Figure S2: effect of Nrf2 on the NLRP3 inflammasome downstream production IL-1*β* and IL-18 releases after ICH. Figure S3: effect of Nrf2 on the activation of caspase-1 after ICH.
