# Supplementary figures and images for: iTRAQ-Based Quantitative Proteomics Indicated Nrf2/OPTN-Mediated Mitophagy Inhibits NLRP3 Inflammasome Activation after Intracerebral Hemorrhage
Source: Oxid Med Cell Longev. 2021 Feb 9;2021:6630281. doi: 10.1155/2021/6630281 (PMC7892225; doi:10.1155/2021/6630281)

## Nrf2

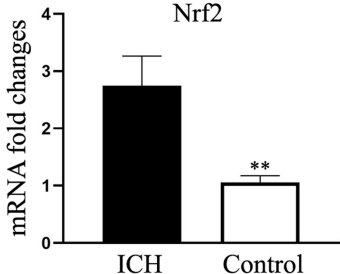

## NLRP3

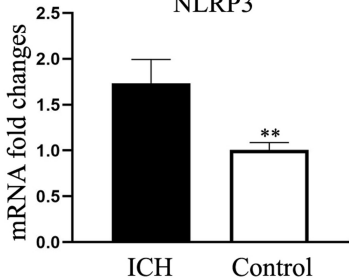

Supplement: Supplementary Materials — Figure S1: the fold changes of Nrf2 and NLRP3 in ICH and control groups. Figure S2: effect of Nrf2 on the NLRP3 inflammasome downstream production IL-1β and IL-18 releases after ICH. Figure S3: effect of Nrf2 on the activation of caspase-1 after ICH. [file 6630281.f1.zip › Figure S1.pdf]

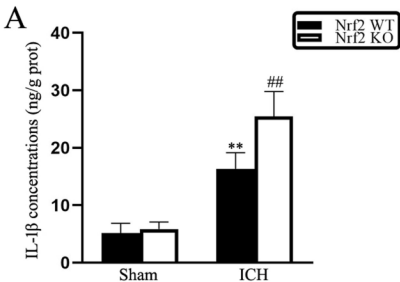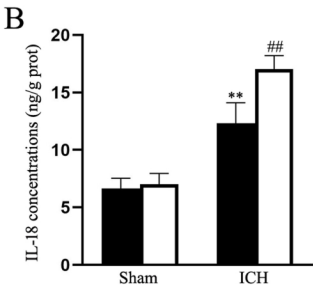

Supplement: Supplementary Materials — Figure S1: the fold changes of Nrf2 and NLRP3 in ICH and control groups. Figure S2: effect of Nrf2 on the NLRP3 inflammasome downstream production IL-1β and IL-18 releases after ICH. Figure S3: effect of Nrf2 on the activation of caspase-1 after ICH. [file 6630281.f1.zip › Figure S2.pdf]

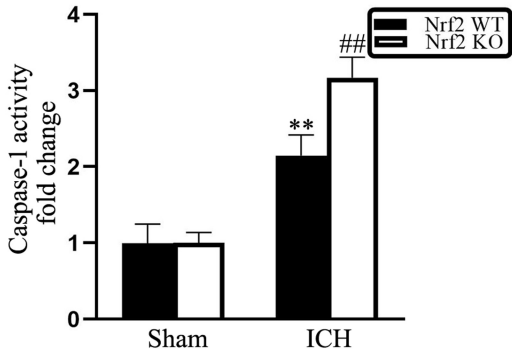

Supplement: Supplementary Materials — Figure S1: the fold changes of Nrf2 and NLRP3 in ICH and control groups. Figure S2: effect of Nrf2 on the NLRP3 inflammasome downstream production IL-1β and IL-18 releases after ICH. Figure S3: effect of Nrf2 on the activation of caspase-1 after ICH. [file 6630281.f1.zip › Figure S3.pdf]
